# Combining X-ray excited optical luminescence and X-ray absorption spectroscopy for correlative imaging on the nanoscale

**DOI:** 10.1107/S1600577521009450

**Published:** 2021-11-03

**Authors:** Selwin Hageraats, Katrien Keune, Stefan Stanescu, Jean-Michel Laurent, William Fresquet, Mathieu Thoury

**Affiliations:** a Conservation and Science, Rijksmuseum Amsterdam, PO Box 74888, 1070 DN Amsterdam, The Netherlands; b IPANEMA, CNRS, Ministère de la Culture et de la Communication, Université de Versailles Saint-Quentin-en-Yvelines, USR 3461, Université Paris-Saclay, 91128 Gif-sur-Yvette, France; c Van ’t Hoff Institute for Molecular Science, University of Amsterdam, PO Box 94157, 1090 GD Amsterdam, The Netherlands; d Synchrotron SOLEIL, 91192 Gif-Sur-Yvette, France; e Andor Technology, Springvale Business Park, 7 Millennium Way, Belfast BT12 7AL, United Kingdom

**Keywords:** XEOL, STXM, soft X-rays, ZnO, XANES

## Abstract

X-ray absorption and optical luminescence can both provide valuable but very different information on the chemical and physical properties of materials. Although it is known that the spectral characteristics of many materials are highly heterogeneous on the micro- and/or nanoscale, no methodology has so far been shown to be capable of spatially resolving both full X-ray absorption and X-ray excited optical luminescence (XEOL) spectra on the nanoscale in a correlative manner. For this purpose, the scanning transmission X-ray microscope at the HERMES beamline of the SOLEIL synchrotron was equipped with an optical detection system capable of recording high-resolution XEOL spectra using a 40 nm soft X-ray probe. The functionality of the system was demonstrated by analyzing ZnO powder dispersions — showing simultaneously the X-ray linear dichroism and XEOL behavior of individual submicrometric ZnO crystallites.

## Abstract 2

A multimodal spectral imaging technique is demonstrated that allows simultaneous acquisition of soft X-ray transmission and X-ray excited optical luminescence. The use of a Fresnel zone plate ensures a lateral resolution of the correlative spectral images of approximately 40 nm.

## 1. Introduction

X-ray excited optical luminescence (XEOL) is a phenomenon that been known and used since the discovery of X-rays. Initially, the phenomenon was primarily exploited as a means to detect and visualize X-rays, as is still the case today. The analytical applications of XEOL were not explored until the 1960s, when XEOL began being used for the detection of rare-earth traces (Makovsky *et al.*, 1962 ▸; Linares *et al.*, 1965 ▸). Further studies in the 1970s explored the applicability of XEOL for the detection of trace gases, and the ability to detect and identify carcinogenic polynuclear aromatic hydro­carbons (PAH) in coal (Goldstein *et al.*, 1974 ▸; Woo *et al.*, 1978 ▸).

In recent years, the developments of synchrotron light sources and ultrafast light detectors have triggered a plethora of new studies in which XEOL is performed in combination with X-ray absorption spectroscopy (XAS) to study materials in a highly site-selective manner. Tuning the X-ray energy to diagnostic electronic core-level transitions, XEOL can be induced selectively on specific material phases (Hessel *et al.*, 2008 ▸; Sham *et al.*, 2004 ▸; Armelao *et al.*, 2007 ▸; Kim *et al.*, 2007 ▸; Wang *et al.*, 2014*a* ▸). Alternatively, the intensity of specific XEOL emission bands can be recorded as a function of the X-ray excitation energy, thus obtaining site-selective X-ray absorption spectra (Sham *et al.*, 2004 ▸; Armelao *et al.*, 2007 ▸; Kim *et al.*, 2007 ▸; Wang *et al.*, 2014*a* ▸,*b* ▸; Soderholm *et al.*, 1998 ▸; Rosenberg *et al.*, 2005 ▸; Liu *et al.*, 2010 ▸; Dalba *et al.*, 1999 ▸; Sham & Gordon, 2010 ▸). Such XAS–XEOL studies are primarily focused on semiconductor structures, in which defects and/or surface states exhibit distinctive luminescence and correlations to specific chemical phases can be established based on characteristic X-ray absorption spectra. The thus obtained information can help in the design of optical materials — especially those for which luminescent transitions may serve a purpose in biological markers or light-emitting devices.

Besides the previously discussed reports of XAS–XEOL spectroscopy, a multitude of studies have been focused on developing imaging applications based on XEOL. The three main motivations for exploiting XEOL in imaging studies are its low background signal and high penetrability in biological systems, the previously discussed site-selectivity, and the ability to probe optical luminescence at scales below the diffraction limit. These motivations have led to the development of laboratory-based, low-dose, 2D and 3D imaging systems for monitoring drug release and biomolecular processes *in vivo* (Pratx *et al.*, 2010 ▸; Chen *et al.*, 2011 ▸, 2013 ▸; Carpenter *et al.*, 2012 ▸), full-field optically detected XAS microscopes (Poolton *et al.*, 2006 ▸; Sabbe *et al.*, 2014 ▸), and scanning XEOL microscopes that make use of soft and hard X-ray nanoprobes (Sham *et al.*, 2010 ▸; Jacobsen *et al.*, 1993 ▸; Martínez-Criado *et al.*, 2006 ▸, 2012*a* ▸,*b* ▸, 2017 ▸; Wang *et al.*, 2018 ▸).

Although some previously referenced papers show that the implementation of XEOL coupled to synchrotron light sources does allow for the simultaneous detection of full XAS and XEOL spectra, this capability has so far not been reported in a microscopy application. However, for the analysis of chemically heterogeneous materials, probing XAS and XEOL on the bulk of the material rarely provides an accurate representation of the actual material properties. For this reason, a methodology was developed that is capable of performing correlative XAS–XEOL nanoimaging by combining soft X-ray scanning transmission microscopy (STXM) with a sensitive wavelength-dispersed optical detection system. This work describes the instrumental set-up and reports the first results obtained on ZnO powders produced through the metal vapor oxidation synthesis method. This newly developed imaging methodology is anticipated to provide unique and fundamental insights into the correlation between optical transitions and their origin in the different phases of heterogeneous materials.

## 2. Methods

### 2.1. Sample preparation

Samples from two different batches of ZnO were analyzed. The first sample (hereafter labeled ZnO-W) was obtained from an industrial manufacturer and was originally produced to be used as the pigment zinc white. The second sample (hereafter labeled ZnO-L) was laboratory-synthesized by combusting Zn-foil (high purity 99.99%, 0.125 mm thickness; Advent Research Materials Ltd) in an airtight stainless-steel glovebox. After three cycles of outgassing and purging with Ar, an 80/20 mixture of Ar and O_2_ (Air Liquide, purity Ar > 99.99% and O_2_ > 99.995%) was introduced into the glovebox to a total pressure of 1 bar. After combustion of the Zn-foil in this controlled atmosphere, the ZnO powder was collected in the inert atmosphere on a glass plate. X-ray photoelectron spectroscopy (XPS) analysis of this laboratory-synthesized batch showed no detectable impurities.

The two ZnO powders were prepared for XAS–XEOL analysis by dispersing <1 mg in a few millilitres of iso­propanol and sonicating for 30 min to obtain a suspension that appears only slightly hazy to the naked eye. Approximately 5 µL of the suspension was then drop-casted on a 100 nm thin SiN window that was heated to ∼100°C. Within several seconds, all the iso­propanol evaporated, leaving a small amount of powder dispersed on the window surface.

### 2.2. Correlative XAS–XEOL nanoimaging

XAS–XEOL experiments were conducted at the HERMES beamline at the SOLEIL synchrotron. The STXM (Research Instruments GmbH) was equipped with a 30 nm outer ring width Fresnel zone plate (FZP) lens and 50 µm order-sorting aperture to focus the monochromated, linearly polarized beam down to a diameter of approximately 40 nm. SiN windows supporting the various zinc white samples were fixed to an *XYZ* scanning stage, *Z* being the direction collinear with the X-ray beam propagation and used to bring the sample onto the FZP focal plane. STXM images were obtained by raster scanning the *XY* position and recording the transmitted photons using a photomultiplier tube (PMT). Based on low-resolution overview images of the SiN windows, the ZnO dispersions were found to consist of numerous clusters of several tens to hundreds of mostly unstacked ZnO crystallites — each cluster measuring several micrometres in diameter. Therefore, the dimensions of the STXM images were set to be in the range 3 µm × 3 µm to 6 µm × 6 µm.

**Figure 1:**
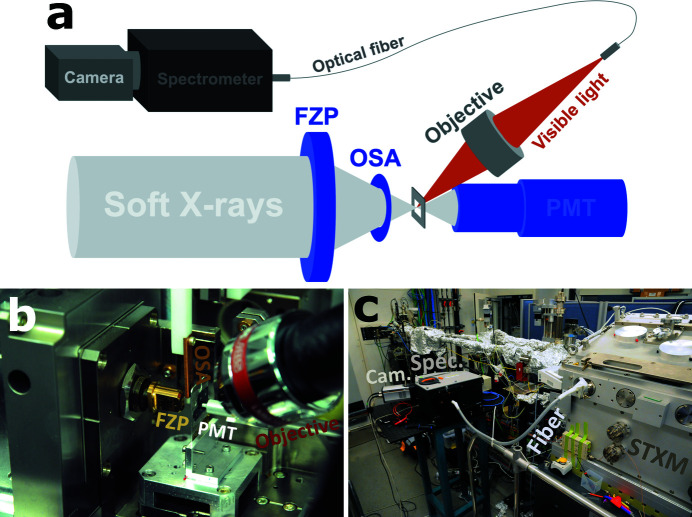
Schematic representation (*a*) and photographs (*b*, *c*) of the XEOL set-up, as implemented in the STXM at the HERMES beamline at Synchrotron SOLEIL.

XEOL analysis was performed within the STXM set-up, using a silver-coated 15× reflective objective with a numerical aperture (NA) of 0.3 (Thorlabs, Inc.) to collect the optical luminescence and focus it into a 7 µm × 200 µm round-to-linear high-hydroxyl content optical fiber bundle (Thorlabs, Inc.). A photograph and schematic depiction of the set-up is shown in Fig. 1 ▸. The optical fiber was fed through the walls of the experimental chamber using a home-made vacuum-sealed feedthrough. Due to small air leaks in the optical fiber, the pressure in the experimental chamber could only be maintained at pressures between 0.2 and 0.7 mbar. The optical fiber was coupled into an Andor Kymera 328i spectrograph which was read out using an Andor Newton 970 EMCCD, operated in regular CCD mode, binning eight pixel columns per spectral channel. Hyperspectral XEOL maps were recorded by operating the STXM in its regular raster scanning mode, but with the upstream slits opened to enable the highest photon flux onto the sample. By using the STXM clock as an external trigger for CCD acquisition, series of XEOL spectra could be recorded and reconstructed to hyperspectral maps.

Since running the STXM with the upstream slits opened may cause damage to the PMT, the choice was made here to record the XEOL and STXM maps separately, but on the same sample regions. Therefore, to achieve correlative maps of XEOL and XAS, ZnO crystallite clusters were first localized by operating the STXM with the PMT switched on and the upstream slits narrowed, after which the PMT was switched off, the slits opened, and the area scanned whilst triggering the acquisition of the CCD camera. Finally, the slits are narrowed again, the PMT switched on, and the area scanned again for a number of X-ray energies.

### 2.3. Data processing

The hyperspectral XEOL data were transformed into false-color maps by integrating the emission bands and assigning each matrix to either the red (band gap emission, 362–402 nm), green (green trap state emission) and blue (blue trap state emission) RGB channel. Integration boundaries for the green and blue trap state emission were determined separately for the two samples, as it was found that the centers of these emission bands differed substantially.

STXM data were aligned using a Fourier alignment algorithm and transformed from transmission to optical density (OD) in the *aXis2000* software. As there is no clear contrast in X-ray absorption behavior between individual ZnO crystallites other than that caused by ZnO’s X-ray natural linear dichroism (XNLD) properties, X-ray absorption contrast was here visualized according to the principles laid out by Hageraats *et al.* (2021 ▸). In the first approach, full Zn *L*-edge X-ray absorption near-edge structure (XANES) spectra are recorded, after which the XNLD orientation contrast is visualized using the SiVM-NNLS method that was adapted from the *DataHandlerP* software and is described in more detail by Hageraats *et al.* (2021 ▸). In the resulting maps, the red and the blue color represent different orientations of the ZnO crystallites with respect to the polarization angle of the X-ray excitation beam. In the remainder of this article, this approach will be referred to as *many-energy* STXM. In the second approach, STXM maps are recorded at only two Zn *L*-edge energies (1031 and 1033 eV) that have been shown to be diagnostic for the XNLD effect in ZnO. Recording maps of both energies with a linear horizontal and linear vertical polarization, calculating for each pixel the following values,

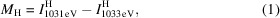




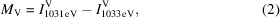

and assigning the resulting matrices to the blue and red channel, respectively, then produces false-color XNLD orientation contrast maps. In the remainder of this article, this approach will be referred to as *two-energy* STXM.

## 3. Results

**Figure 2:**
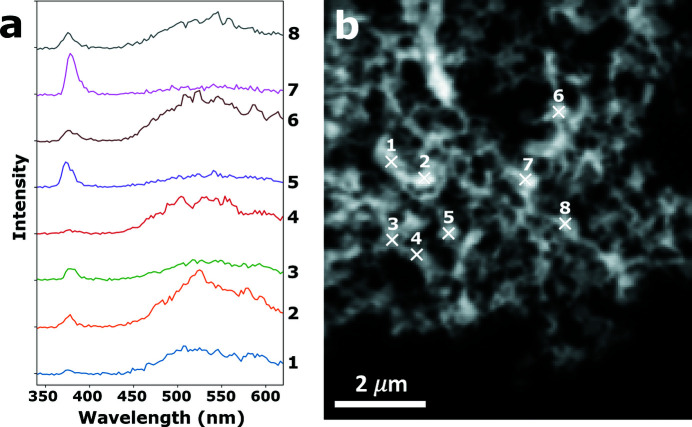
XEOL point spectra recorded on the ZnO-W sample (1050 eV excitation energy) (*a*) and a STXM reference image that shows the corresponding locations on a ZnO crystallite agglomerate (1030 eV excitation energy) (*b*).

To test whether the optical detection set-up was sensitive enough to detect the weak XEOL signal, point spectra were first recorded on the ZnO-W sample, with the X-ray beam tuned to 1050 eV (close to the absorption maximum of the Zn *L*-edge). A *many-energy* STXM reference image and the corresponding XEOL spectra (10 s integration time) are shown in Fig. 2 ▸. The two emission bands corresponding to band gap emission and green trap state emission can clearly be distinguished around 380 and 530 nm, respectively. The considerable variation in the ratio of the two emission bands is in line with previous observations by Bertrand *et al.* (2013 ▸) and makes for a suitable sample to demonstrate the ability of STXM-coupled XEOL to resolve emission contrast at the nanoscale. An integration time of 10 s produces spectra with several hundred counts per energy channel and between 10^3^ to 10^4^ counts per emission band. This spectral quality is deemed more than sufficient to produce maps that show the emission behavior per crystallite. As a compromise between measurement speed and data quality, integration times were therefore chosen between 1 and 5 s for the recording of the hyperspectral XEOL maps, depending on the luminescent intensity of the sample and the purpose of the measurement.

**Figure 3:**
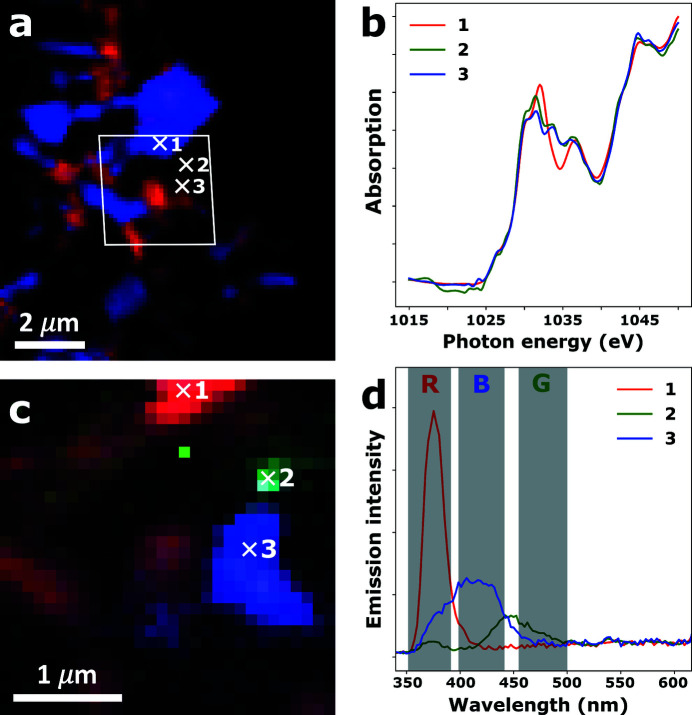
Correlative XAS–XEOL images recorded on a ZnO crystallite agglomerate of the ZnO-L sample. (*a*) False-color *many-energy* STXM image showing XNLD orientation contrast retrieved through SiVM-NNLS analysis. The white parallelogram indicates the region at which XEOL data were recorded. (*b*) Zn *L*-edge XANES retrieved from the *many-energy* STXM map at the locations indicated by the white crosses. (*c*) False-color XEOL image, obtained by integrating the band gap emission (352–390 nm, red channel), green trap state emission (455–500 nm, green channel) and blue trap state emission (399–442 nm, blue channel) collected with the excitation energy tuned to 1050 eV. (*d*) XEOL spectra retrieved from the hyperspectral XEOL map at the locations indicated by the white crosses.

To demonstrate the *many-energy* STXM approach to recording correlative XAS–XEOL images, a full data set recorded on sample ZnO-L is shown in Fig. 3 ▸. From the false-color STXM and XEOL images shown in Figs. 3 ▸(*a*) and 3(*c*), respectively, it is clear that single ZnO crystallites can be located by both techniques, allowing both Zn *L*-edge XANES and XEOL spectra to be retrieved on the very same single particles. Three particles were selected for which the full Zn *L*-edge XANES and XEOL spectra are shown in Figs. 3 ▸(*b*) and 3(*d*). The Zn *L*-edge XANES clearly show the XNLD behavior discussed by Hageraats *et al.* (2021 ▸), with the intensities of the transitions at 1031 and 1033 eV exhibiting a negative correlation. In the XEOL spectra, three distinct absorption bands can be distinguished around 380 nm (band gap emission), around 415 nm (blue trap state emission) and around 450 nm (blue–green trap state emission).

**Figure 4:**
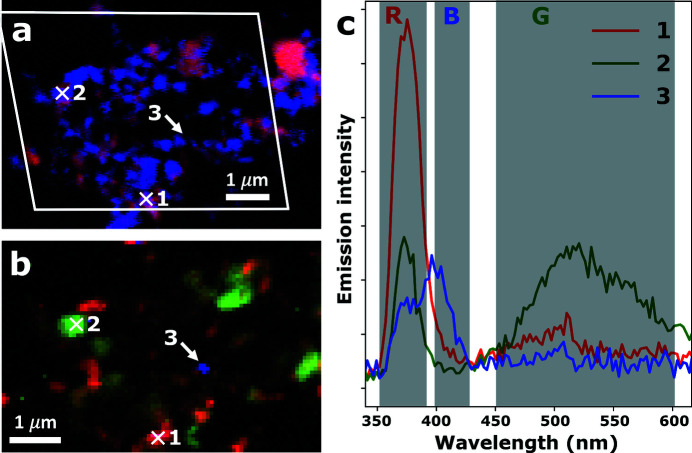
Correlative XAS–XEOL images recorded on a ZnO crystallite agglomerate of the ZnO-W sample. (*a*) False-color *two-energy* STXM image showing XNLD orientation contrast. The white parallelogram indicates the region at which XEOL data were recorded. (*b*) False-color XEOL image, obtained by integrating the band gap emission (352–390 nm, red channel), green trap state emission (451–601 nm, green channel) and blue trap state emission (399–427 nm, blue channel) collected with the excitation energy tuned to 1050 eV. (*c*) XEOL spectra retrieved from the hyperspectral XEOL map at the locations indicated by the white crosses and the white arrow.

The *two-energy* approach to recording correlative XAS–XEOL images is shown in Fig. 4 ▸. This data set was recorded on the ZnO-W sample, which can be seen to exhibit XEOL bands that are distinctly different from those observed on the ZnO-L sample [Fig. 3 ▸(*d*) versus Fig. 4 ▸(*c*)]. Not only is the blue emission band far less common and the green emission band far more common in sample ZnO-W, the two emission bands also appear at different wavelengths: 415 and 450 nm for ZnO-L versus 400 and 520 nm for ZnO-W. As was the case for the XAS–XEOL images shown in Fig. 3 ▸, most of the single ZnO crystallites shown in Figs. 4 ▸(*a*) and 4(*b*) can readily be correlated.

The hyperspectral XEOL maps shown in Figs. 3 ▸ and 4 ▸ were recorded using 5 and 2 s integration times, respectively, allowing about 10^3^ spectra to be recorded per hour. For samples that exhibit heterogeneity in XEOL behavior similar to that observed on the ZnO-L and ZnO-W samples, it is generally preferable to be able to analyze several tens to several hundreds of particles — thereby increasing the statistical significance of any findings. Early (unrecorded) observations of decreasing XEOL signal for repeated spectral measurements at single ZnO crystallites suggested that the high photon flux hitting the sample may induce a form of photobleaching. If photobleaching indeed occurs, the efficiency of the XEOL process may rapidly decrease, putting a practical upper limit on the total signal that can be collected per crystallite. Although this is problematic for XEOL studies in which a high signal-to-noise (SNR) ratio is desired, it could also mean that the integration times reported in this study can be reduced considerably without substantial compromise to the total signal collected.

**Figure 5:**
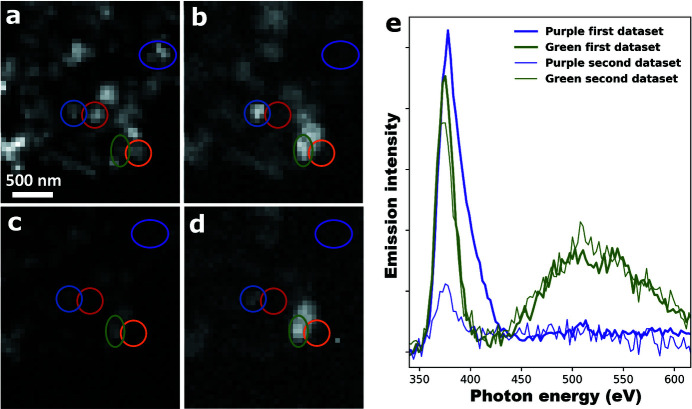
Images showing the integrated intensities of the band gap emission (*a*, *c*) and green trap state emission (*b*, *d*) for two consecutive hyperspectral XEOL maps, each recorded with 1 s integration time per spectrum and an excitation energy of 1050 eV. The colored ovals indicate specific ZnO crystallites that are discussed in the text. (*e*) Average XEOL spectra for the ZnO crystallites circled in purple and green, taken from the two consecutive hyperspectral XEOL maps.

In order to quantify the photobleaching effect on ZnO, two consecutive hyperspectral XEOL maps were recorded on a ZnO crystallite agglomerate of the ZnO-W sample — each with 1 s integration time per spectrum. Images of the integrated intensities of the band gap and green emission for both consecutive XEOL maps are shown in Fig. 5 ▸. Comparing the intensities for the first and second XEOL map, it can be seen that the signal recorded in the first is generally two to three times higher than the signal recorded in the second. However, there is significant heterogeneity in the extent to which the ZnO crystallites are photobleached. For instance, the crystallite circled in purple experienced a very significant drop in intensity, whereas the emission intensity of the crystallite circled in green appears to have not changed at all. Here, it is interesting to note that the crystallites that exhibit the most intense green emission initially, appear to experience the smallest drop in emission intensity upon X-ray irradiation.

## 4. Discussion

From the results shown in Figs. 2 ▸, 3 ▸ and 4 ▸, it can be concluded that the XAS–XEOL set-up indeed allows to perform correlative nanoimaging, providing for each spatial point full XEOL spectra and either full Zn *L*-edge XANES or information on selected diagnostic Zn *L*-edge transitions. Still, the results reported here were obtained on the semiconductor ZnO, which, due to its direct optical band gap, generally has a high luminescence yield. Applications may also be envisioned that involve more weakly luminescing compounds or require the acquisition of a far greater number of spatial points. In such cases, the current set-up may not suffice and improvements to the optical detection system need to be considered.

The main factor determining whether or not application of the technique is feasible is the number of emitted photons that can be made to reach the CCD detector and transferred to digital counts per unit time. This number depends on the flux of X-ray photons hitting the sample, the efficiency of the XEOL process, the collection solid angle, the efficiency of focusing the collected light into the optical fiber cores, the overall efficiency of the spectrometer optics, and the quantum efficiency (QE) of the CCD detector. For the set-up described here, the combined efficiency of the optical set-up is of the order of 10^−3^, meaning that, for every 10^3^ photons emitted by the sample, only one count is registered by the CCD. As the combined efficiency of the spectrometer–detector system is already close to what is technologically feasible, the most substantial improvements can be realized by increasing the collection solid angle. It can be seen in Fig. 1 ▸(*b*) that the use of objectives with larger NAs is not permitted due to the proximity of the PMT. However, optical alignments can be envisioned that include two, or even as many as four, identical 0.3NA reflective objectives. Depending on the number of additional objectives and whether CCD read-out noise or shot noise is dominant, an increase of the SNR by a factor of 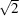 to 4 can be realized for a given exposure time.

Another way in which the current set-up could be improved upon is by fully synchronizing the XEOL and XAS acquisition. Currently, the two acquisitions are performed successively so as to permit XEOL to be recorded with a photon flux that is likely to be higher than the damage threshold for the PMT. By fixing an intensity filter in front of the PMT (*e.g.* thin aluminium foil), it will be possible to operate the STXM with the upstream slits opened, while still being able to safely record X-ray absorption. With such a synchronized collection approach, it is preferable to limit the acquisition of X-ray absorption data to only a few energies, so as to prevent each XEOL spectrum being made up of many single acquisitions — thereby accumulating excessive amounts of read-out noise. Especially for compounds that undergo photooxidation or photoreduction reactions when exposed to high-intensity X-ray beams, this synchronized approach can both limit the total measurement time per sample and (at least partially) prevent X-ray exposure during XEOL acquisition to affect the X-ray absorption characteristics.

Besides being a problematic side-effect of performing XEOL measurements using a high-brilliance X-ray beam, the previously described photobleaching effect also provides unique information on the relationship between crystallinity and luminescence emission intensity. Here, it is relevant to refer back to Hageraats *et al.* (2021 ▸), in which it was unequivocally shown that there is a significant effect of high X-ray radiation doses (and dose rates) on the crystallinity of ZnO. The decrease in XNLD properties shown by Hageraats *et al.* (2021 ▸) [Figs. 2 ▸(*e*)–2(*h*) therein and quantified in their Tables 1 and 2] was the result of approximately 600 ms of exposure per spatial point. It is therefore reasonable to assume that the decrease in XEOL intensity shown in Fig. 5 ▸ — induced by 1 s of beam exposure — is related to a decrease in crystalline ordering of the ZnO crystallites. Whether or not the decrease in crystalline ordering is a *direct* cause of the observed photobleaching effect can be derived from previous reports on the photoluminescence behavior of amorphous ZnO. Most importantly, it has been shown that amorphous ZnO — like crystalline ZnO — also exhibits UV luminescence and that its intensity can be higher than for similarly produced crystalline ZnO (Eo *et al.*, 2010 ▸; Zhang *et al.*, 2007 ▸). Therefore, it is proposed that the decrease in band gap emission is not a *direct* result of reduced crystallinity, but is rather caused by the introduction of non-radiative recombination centers within the band gap.

An interesting observation in this context is that, for the ZnO crystallites for which the decrease in band gap emission intensity is the strongest (three cases indicated by the red, orange and purple circles in Fig. 5 ▸), no green trap state emission was observed. This observation suggests two possible radiation damage mechanisms. First, the focused X-ray beam may heat the ZnO crystallite, the dissipation of which depends on the specific surface area. As it is often hypothesized that the green trap state emission is related to intrinsic surface defects (Gong *et al.*, 2007 ▸; Röhr *et al.*, 2019 ▸; Zhou *et al.*, 2007 ▸), the green emission shown in Fig. 5 ▸(*b*) may be a good measure of the crystallite to dissipate the radiation-induced heat. Second, intense X-ray irradiation of ZnO can cause excessive population of the mostly antibonding conduction band (Ivanov & Pollmann, 1981 ▸), causing structural deformation and eventually permanent structural damage. In that case, higher concentrations of de-excitation pathways could alleviate the radiation damage. As green trap state emission suggests a non-stoichiometric ZnO crystallite with ubiquitous recombination centers, the intensity of green XEOL could indeed be a measure of its radiation damage resistance. Based on the approximate photon flux at the sample (>10^8^ photons s^−1^), the approximate dimensions of the ZnO crystallites (∼200 nm), and the heat capacity of ZnO (40.3 J K^−1^ mol^−1^), the energy absorbed by a ZnO crystallite per second during XEOL analysis is enough to heat it up by 10^6^ K. As this estimation suggests an important role for heat dissipation in preventing radiation damage, the first hypothesis is deemed most likely to be true.

## 5. Conclusion

This research paper describes how soft X-ray XAS and XEOL can be recorded in a correlative manner on the nanoscale by coupling an optical detection system to STXM. Through analysis of two differently produced ZnO powders it was shown that full spectral XEOL and Zn *L*-edge XANES could be correlated at an approximate lateral resolution of 40 nm — well below the diffraction limit for near-UV and visible light. The results show a clear ability to determine the XEOL emission behavior of individual crystallites, determine accurately the positions of the various emission bands, and correlate these measures to their *many-energy* or *two-energy* X-ray absorption behavior. Due to the use of a tightly focused, high-brilliance X-ray beam, the samples experienced significant radiation damage — shown by Hageraats *et al.* (2021 ▸) to introduce a degree of amorphism into the ZnO crystallites. In terms of XEOL behavior, the radiation damage causes a heterogeneously distributed decrease in both band gap and green trap emission. The decrease in band gap emission intensity was found to be negatively correlated to the intensity of green emission prior to irradiation — an effect that can likely be ascribed to the faster heat dissipation of particles with a larger specific surface area. Given the potential improvements in the set-up’s collection solid angle by a factor of two to four and the ability to decrease the integration time without compromising much on the SNR, substantial improvements could still be realized in terms of measurement speed. In that case, the correlative XAS–XEOL nanoimaging methodology could find wide application for studies of heterogeneous materials, even if they do not luminesce as brightly as ZnO.
